# NDV-induced autophagy enhances inflammation through NLRP3/Caspase-1 inflammasomes and the p38/MAPK pathway

**DOI:** 10.1186/s13567-023-01174-w

**Published:** 2023-06-05

**Authors:** Juncheng Cai, Siyuan Wang, Haoyun Du, Lei Fan, WeiFeng Yuan, Qiufan Xu, Jinlian Ren, Qiuyan Lin, Bin Xiang, Chan Ding, Tao Ren, Libin Chen

**Affiliations:** 1 College of Veterinary Medicine, South China Agricultural University, Guangzhou, 510642 China; 2 National and Regional Joint Engineering Laboratory for Medicament of Zoonosis Prevention and Control, Guangzhou, China; 3 Key Laboratory of Animal Vaccine Development, Ministry of Agriculture, Guangzhou, China; 4 Key Laboratory of Zoonosis Prevention and Control of Guangdong Province, Guangzhou, China; 5 College of Veterinary Medicine, Yunnan Agricultural University, Kunming, China; 6 Shanghai Veterinary Research Institute, Chinese Academy of Agricultural Sciences, Shanghai, 200241 China

**Keywords:** Autophagy, inflammation, mitochondrial damage, mitophagy, Newcastle disease virus

## Abstract

Newcastle disease (ND), caused by the Newcastle disease virus (NDV), is a highly virulent infectious disease of poultry. Virulent NDV can cause severe autophagy and inflammation in host cells. While studies have shown a mutual regulatory relationship between autophagy and inflammation, this relationship in NDV infection remains unclear. This study confirmed that NDV infection could trigger autophagy in DF-1 cells to promote cytopathic and viral replication. NDV-induced autophagy was positively correlated with the mRNA levels of inflammatory cytokines such as IL-1β, IL-8, IL-18, CCL-5, and TNF-α, suggesting that NDV-induced autophagy promotes the expression of inflammatory cytokines. Further investigation demonstrated that NLRP3 protein expression, Caspase-1 activity, and p38 phosphorylation level positively correlated with autophagy, suggesting that NDV-induced autophagy could promote the expression of inflammatory cytokines through NLRP3/Caspase-1 inflammasomes and p38/MAPK pathway. In addition, NDV infection also triggered mitochondrial damage and mitophagy in DF-1 cells, but did not result in a large leakage of reactive oxygen species (ROS) and mitochondrial DNA (mtDNA), indicating that mitochondrial damage and mitophagy do not contribute to the inflammation response during NDV infection.

**Supplementary Information:**

The online version contains supplementary material available at 10.1186/s13567-023-01174-w.

## Introduction

Newcastle disease (ND) is an acute, febrile, and highly contagious disease caused by virulent strains of Newcastle disease virus (NDV) in a variety of birds, especially chickens and turkeys. Due to the severe economic losses caused, ND is an acute disease harming the poultry industry [1]. NDV is a member of the *Paramyxoviridae* family with only one serotype, and a single-stranded, non-segmented, negative-sense RNA virus, encoding six major structural proteins: nucleocapsid protein (NP), phosphoprotein (P), matrix protein (M), fusion protein (F), hemagglutinin-neuraminidase (HN), and large polymerase protein (L) in the following order: 3’-NP-P-M-F-HN-L-5’ [2]. However, the pathogenesis of NDV still has many unresolved mysteries, which is a constraint to its prevention and control.

There are three main types of autophagy based on how the cellular contents are incorporated into the lysosome: microautophagy, chaperone-mediated autophagy, and macroautophagy [3]. In addition, autophagy can specifically degrade specific macromolecules or organelles by selective autophagy, such as mitophagy, reticulophagy, and ribophagy [3, 4]. Macroautophagy, usually referred to simply as autophagy, is a ubiquitous physiological phenomenon in eukaryotic cells and an evolutionarily conserved process induced in response to diverse stress stimuli (hypoxia, starvation, toxic molecules, and pathogen invasion) to maintain cell homeostasis. Autophagy comprises four steps: (1) the formation and expansion of the isolation membrane, (2) the encapsulation and phagocytosis of the substrate, (3) the formation of autolysosomes, and (4) substrate degradation within the lysosomes. Several autophagy-related proteins have been implicated in the formation of autophagosomes [3]. Microtubule-associated protein 1 light chain 3 (LC3), the mammalian homolog of yeast autophagy-related gene 8 (Atg8), is the most widely monitored autophagy-related protein [5, 6].

Viral infection and replication cause cellular stress, and autophagy is a frequent outcome of infection [7]. However, autophagy is not merely a passive process during viral infection. Since autophagy mainly involves the degradation and reuse of cytoplasmic components, the innate immune system activates autophagy to degrade invading viruses [8, 9]. Studies have found that while the vesicular stomatitis virus (VSV) has no pathogenic effect on flies, it causes the death of flies lacking Atg [10]. During Sindbis virus (SINV) infection, autophagy protein Beclin 1 protects against SINV-mediated encephalitis, and p62 (also called sequestosome 1 (SQSTM1)) binds to a SINV capsid protein targeting it to the autophagosome [11, 12].

Although autophagy is an important defense mechanism in the host cells, some viruses have evolved strategies to escape or inhibit multiple steps of the autophagic pathway, making it even more beneficial for their survival in some cases [9]. For example, viral proteins of human cytomegalovirus (HCMV), TRS1, and IRS1 block autophagy by interacting with Beclin 1 [13, 14]. Similarly, Herpes simplex virus 1 (HSV-1) encoding neurovirulence factor ICP34.5 also interacts with Beclin 1 to inhibit autophagy [15]. Some γ-herpesviruses directly interact with Beclin 1 via the Bcl-2 homologs [16, 17]. Moreover, some studies have provided strong evidence that NDV infection induces autophagy, promoting NDV replication [18]. However, the specific mechanism remains unknown.

Inflammation underlies a wide variety of physiological and pathological processes. Controlled inflammatory responses protect against infection but can be detrimental if dysregulated. During the immunological processes, toll-like receptors (TLRs) and NOD-like receptors (NLRs) recognize pathogen-associated molecular patterns (PAMPs) and damage-associated molecular patterns (DAMPs), activating immune cells, promoting the secretion of proinflammatory cytokines and stimulating inflammatory response [19, 20]. We have previously shown a significant increase in IL-1β gene expression in SPF chickens and DF-1 cells infected with virulent NDV strain [21]. Moreover, neutralizing IL-1β significantly reduced chicken morbidity and mortality, suggesting that NDV-induced inflammation plays a vital role in the pathogenesis of NDV infection and death [22, 23]. Hence, it is necessary to clarify the mechanism underlying NDV-induced inflammation, to reduce the resultant inflammatory damage.

Autophagy is induced during the activation of an innate immune response and affects inflammation by negatively regulating TLR and NLR signaling, creating feedback loops between autophagy and inflammation [24, 25]. Additionally, mitochondria also connect autophagy and inflammation during viral infections [26–28]. Some viruses have evolved to exploit the relationship between autophagy and inflammation to promote viral replication [8, 29]. Zhang et al. demonstrated that the autophagy inhibitors Wort and CQ reduce Japanese encephalitis virus (JEV) infection and weaken the inflammatory response in mice [30]. Avian influenza virus (H5N1), another single-stranded RNA virus, was shown to induce autophagy to promote animal lung inflammation through NF-κB and p38/MAPK pathways [31]. Therefore, a mutual regulatory relationship is presumed between autophagy and inflammation during viral infection. However, this relationship remains unknown in NDV.

This study demonstrates that NDV infection triggers autophagy in DF-1 cells, promoting the expression of inflammatory cytokines. By regulating the autophagy pathway using pharmacological inhibitors and promoters, we have identified specific molecules and pathways targeted during autophagy to regulate the expression of inflammatory cytokines during NDV infection. Moreover, we also demonstrate that mitochondrial damage and mitophagy caused by NDV infection have little effect on inflammation response.

## Materials and methods

### Cells, viruses, and plasmids

DF-1 chicken fibroblast cells were purchased from the American Type Culture Collection (ATCC; Manassas, VA). Chicken embryo fibroblast (CEF) primary cells were prepared from 9-day-old specific-pathogen-free (SPF) embryonated chicken eggs. NDV virulent strain GM, Dsred-mito-EGFP and Dsred-LC3-EGFP recombinant plasmids were stored in our laboratory.

### Reagents and antibodies

Rapamycin (R0395), chloroquine (CQ) (C6628), and rabbit polyclonal anti-LC3B antibody (L7543) were purchased from Sigma-Aldrich (St. Louis, MO, USA). 3-Methyladenine (3-MA) (HY-19,312) was purchased from MedChemExpress (Monmouth Junction, NJ, USA). Rabbit polyclonal anti-JNK1 antibody (bs-0501R) and rabbit polyclonal anti-phospho-JNK1 antibody (bs-17591R) were purchased from Bioss (Woburn, MA, USA). Rabbit polyclonal anti-p38 antibody (9212), rabbit polyclonal anti-phospho-p38 antibody (9211), rabbit polyclonal anti-p44/42 antibody (9102) and rabbit polyclonal anti-phospho-p44/42 antibody (9101) were purchased from Cell Signaling Technology (Danvers, MA, USA). Rabbit polyclonal anti-GAPDH antibody (ab9485) was purchased from Abcam (Boston, MA, USA). Horseradish peroxidase (HRP)-conjugated goat anti-rabbit secondary antibody was purchased from Jackson ImmunoResearch (West Grove, PA, USA). Rabbit polyclonal antibodies against NDV nucleocapsid protein (NP) and NOD-like receptor thermal protein domain associated protein 3 (NLRP3) were prepared in our laboratory. Recombinant autophagy related protein 16 like protein 1 (ATG16L1) small interfering RNAs (siRNAs), consisting of three target-specific 21-nucleotide siRNAs designed to specifically knock down chicken ATG16L1 gene expression, along with control scrambled siRNA, were designed and synthesized by Sangon Biotech (Shanghai, China).

### Viral infection, drug treatment, and cell viability assay

DF-1 and CEF cells were infected with NDV at a multiplicity of infection (MOI) of 1 at 37 °C. Following a 1-h absorption period, unattached viruses were removed and the cells were then washed three times with phosphate buffered saline (PBS) and cultured in complete medium at 37 °C.

The optimal concentrations of drugs used in this experiment were determined with Cell Counting Kit-8. Briefly, 5 × 10^4^ cells grown in each well of a 96-well plate were treated with each drug at different concentrations in a 100-µL volume for 24 h. CCK8 solution then was added to each well and incubated for 1 h in the dark. Cell viability was determined by measuring the absorbance at 450 nm against the background control. The concentrations tested for CQ were 1, 5, 10, 25, and 50 µM; those for 3-MA were 1, 5, 7.5, 10, and 15 mM; and those for rapamycin were 10, 15, 25, 50, and 100 µM. The optimal experimental concentration of CQ is 10 µM, 3-MA is 5mM and rapamycin is 15 µM. The drugs were dissolved in dimethyl sulfoxide (DMSO) and diluted in complete medium, respectively. DF-1 or CEF cells were pretreated with optimal concentrations of CQ, 3-MA, or rapamycin for 4 h prior to viral infection. Subsequently, the cells were infected with NDV at an MOI of 1 as described above [18].

### Transmission electron microscopy (TEM)

Transmission electron microscopy was used for observation of autophagy and mitophagy. Specifically, DF-1 or CEF cells were infected with NDV at an MOI of 1, collected at 12 h post-infection, and subjected to preparation of samples for TEM observation. Images of the ultrathin sections were acquired using a CM-120 transmission electron microscope (Philips) at 80 kV.

### Confocal fluorescence microscopy

Confocal fluorescence microscopy was used for analysis of the presentation of LC3 protein and mitochondria in lysosomes after NDV infection. DF-1 cells grown to 60 to 70% confluence on coverslips were transfected with plasmid Dsred-LC3-EGFP or Dsred-mito-EGFP. The cells were treated with virus infection as described above at 24 h post-transfection, and then the fluorescence was visualized with confocal fluorescence microscopy.

### RNA interference

RNA interference was used to knock down ATG16L1 or ATG7, the key genes for autophage formation. ATG16L1-specific silencing RNA (sense: 5′-GCAUGGACCGUAGGGUUAATT-3′; antisense: 5′-UUAACCCUACGGUCCAUGCTT-3′) and ATG7- specific silencing RNA (sense: 5′-GGUGGAAUUAAUGGUUUCUTT-3′; antisense: 5′-AGAAACCAUUAAUUCCACCTT-3′) were designed and synthesized by Sangon Biotech (Shanghai, China). DF-1 cells grown to 60 to 70% confluence in 6-well plates were transfected with ATG16L1 or ATG7-specific Si-RNA or scrambled control (NC) siRNA. Briefly, 200 pmol siRNA was diluted in 120 µL 1×riboFECT™ CP Buffer (Ribobio, Guangdong, China). After incubation for 3 min at room temperature, 12 µL riboFECT™ CP Reagent (Ribobio, Guangdong, China) was added in the diluent and incubated for 20 min at room temperature. The mixtures were then added dropwise to each well. The samples were collected for silencing efficiency tests after incubating at 37 °C for 36 to 48 h.

### Quantitative real-time polymerase chain reaction (qRT-PCR)

Total RNA was extracted from DF-1 cells with NDV infection or drug treatment using TRIzol reagent (Invitrogen, USA); HiScript III RT SuperMix for qPCR (Vazyme, China) was used to remove genomic DNA and reverse transcribe RNA to cDNA. Briefly, 1 µg RNA was added to 4 µL of the 4 × gDNA wiper Mix, and made up to 16 µL by adding RNAse-free water and left for 2 min at 42 ℃ to remove genomic DNA. Then, 4 µL of the 5 × HiScript III qRT SuperMix was added to the reaction mix from the previous step, and left for 15 min at 37 ℃ and 5 s at 85 ℃ to reverse transcribe RNA to cDNA. In order to control for DNA contamination, negative reverse transcription controls were included. We diluted cDNA 10 times for the reaction system in qPCR. We designed primers (Table 1) used for qRT-PCR based on previously reported target sequences [32]. New primers (Table 1) were designed according to the nucleotide sequence of the target gene and synthesized by Sangon Biotech (China). PCR efficiency, linear dynamic range, and primer specificity of newly designed primers (ND1, ND2, ND3, COXI, ATG16L1, and ATG7) had been confirmed (Additional file 1). The mRNA transcription levels of GAPDH were stable across treatments (Additional file 1). The qRT-PCR was performed in a 7500 Fast Real-Time PCR system using SYBR qPCR mix (Vazyme, China), according to the manufacturer’s instructions. Briefly, a master mix consisting of 10 µL 2 × ChamQ Universal SYBR qPCR Master Mix, 7.6 µL RNAse-free water, and 0.2 µL of the specific forward (10 µM) and reverse (10 µM) primers was prepared for one reaction and 2 µL cDNA dilution template was added. For amplification, the following temperature profile was used: 30 s at 95 °C, followed by 40 cycles of 10 s at 95 °C and 30 s at 60 °C, and finally 15 s at 95 °C, 60 s at 60 °C, and 15 s at 95 °C to form the melt curves. The fold change for each gene was calculated using the 2^−ΔΔCT^ method [33].

**Table 1:** **qPCR primers utilized in this study**.

Primer names	Sequence (5′-3′)	GenBank no.	Genome position	
GAPDH-F	CCTCTCTGGCAAAGTCCAAG	NM_204305	120–139	Previous publication
GAPDH-R	CATCTGCCCATTTGATGTTG		300–319	
IL-1β-F	CTGGGCATCAAGGGCTACAA	NM_204524	589–608	
IL-1β-R	CGGTAGAAGATGAAGCGGGT		700–719	
IL-18-F	AGAGCATGGGAAAATGGTTG	NM_204608	530–549	
IL-18-R	TCTTCCTCAAAGGCCAAGAA		678–697	
IL-8-F	CTGGACAAGGCAGACACCAA	NM_205018	362–381	
IL-8-R	AATGGCGTTGTCTCCCACTT		441–460	
CCL-5-F	GGAAGCTGCCCCAGAATCAT	NM_001045832	175–194	
CCL-5-R	GTATTCCTTCACCCACCGGG		289–308	
TNF-α-F	CCGCCCAGTTCAGATGAGTT	AY765397	381–400	
TNF-α-R	GCAACAACCAGCTATGCACC		491–510	
NDV-M-F	AGTGATGTGCTCGGACCTTC	DQ486859	4106–4125	
NDV-M-R	CCTGAGGAGGCATTTGCTA		4205–4225	
NLRP3-F	CGTGTTGGGCAGTTTCACAG	KF318520	1767–1786	
NLRP3-R	GCCCACTGCTTGATGGAGAA		1840–1859	
ND1-F	TTGAGTCCCCCTCCCACTAC	AB753758	106–125	Newly designed
ND1-R	GCTCGAAGGGCTCCGATTAG		236–255	
ND2-F	ACCTTAGCCATCATCCCCCT	KX534729	105–124	
ND2-R	TTAGGCATGATGTCGGGTGG		266–285	
ND3-F	ACGAATGCGGATTTGACCCA	OK077527	10,897–10,916	
ND3-R	TTGGATGGCTCATGGAAGGG		11,009–11,028	
COXI-F	GAACTAGGACAGCCCGGAAC	OK077527	6771–6790	
COXI-R	GGGGAATGCTATGTCTGGGG		6919–6938	
ATG16L1-F	CAAAGAACCCCTGCCTGTTG	XM_004936938	892–911	
ATG16L1-R	AGGGGAGACTCAGACAGACC		1061–1080	
ATG7-F	CTGAGGGGCATGGAGGATGT	NM_001030592	89–108	
ATG7-R	CCACAGGGTTCTGGGACTCA		258–239	

### Reactive oxygen species assay

DCFH-DA is a cellular permeable probe for the detection of intracellular reactive oxygen species (ROS). Briefly, DCFH-DA was dissolved in dimethyl sulfoxide (DMSO) and diluted in complete medium to a concentration of 10 µM. The diluent was added dropwise to each well of cells and incubated for 1 h in dark. Then the DCF fluorescence intensity was measured by flow cytometry.

### Detection of mitochondrial DNA in cytoplasm

Mitochondria were isolated using Mitochondria Isolation Kit for Cultured Cells (Thermo, USA), and total DNA was extracted from the remaining extracts via the Tissue DNA kit (OMEGA, USA). qPCR was performed on extracted DNA using mtDNA primers (*ND-1*, *ND-2*, *ND-3*, and *COXI*), the fold change for each gene was calculated using the 2 − ^ΔΔCT^ method.

### Caspase-1 activity assay

The activity of Caspase-1 in DF-1 cells was measured using a Caspase-1 activity assay kit (Beyotime, Shanghai, China). Cells were digested with trypsin, centrifuged with cell culture supernatants at 1000 rpm for 5 min at 4 °C, and washed once with PBS. Then, the cell pellet was collected, lysed, and centrifuged at 20 000 rpm for 20 min at 4 °C, and the activity of Caspase-1 was determined based on the ability of Caspase-1 to convert acetyl-Tyr-Val-Ala-Asp p-nitroaniline (Ac-YVAD-pNA) into p-nitroaniline (pNA) [23].

### Western blotting

DF1 cells were lysed with RIPA buffer supplemented with PMSF and phosphorylase inhibitors (Beyotime, Shanghai, China), and the protein concentration was tested with Pierce™ BCA Protein Assay Kit (ThermoFisher). Denatured protein was electrophoresed in sodium dodecyl sulfate-polyacrylamide gel electrophoresis gels and transferred to a nitrocellulose membrane (Amersham). After incubation in a blocking buffer (5% nonfat milk powder in Tris-buffered saline containing 0.2% Tween 20 [TBS-T]) for 1 h at room temperature, the membrane was reacted with primary antibodies overnight at 4 °C and HRP-conjugated secondary antibodies for 1 h at room temperature. The membranes were visualized using an Odyssey infrared imaging system (LI-COR Biosciences, Lincoln, NE, USA). Image J software was used to quantify the relative protein levels.

### Statistical analysis

Statistical analyses were performed using GraphPad Prism (version 5.0; GraphPad Software, Inc., La Jolla, CA, USA). Data were expressed as mean ± standard deviation (SD). Data were analyzed using Student *t*-test for pairwise comparisons or an analysis of variance (ANOVA)/Dunn multiple comparison test for multiple comparisons. Statistical significance was set at *P* < 0.05, *P* < 0.01, and *P* < 0.001 for values that were considered significant, very significant, and highly significant, respectively.

## Results

### NDV-induced autophagy promotes cytopathic and viral replication

**Figure 1:**
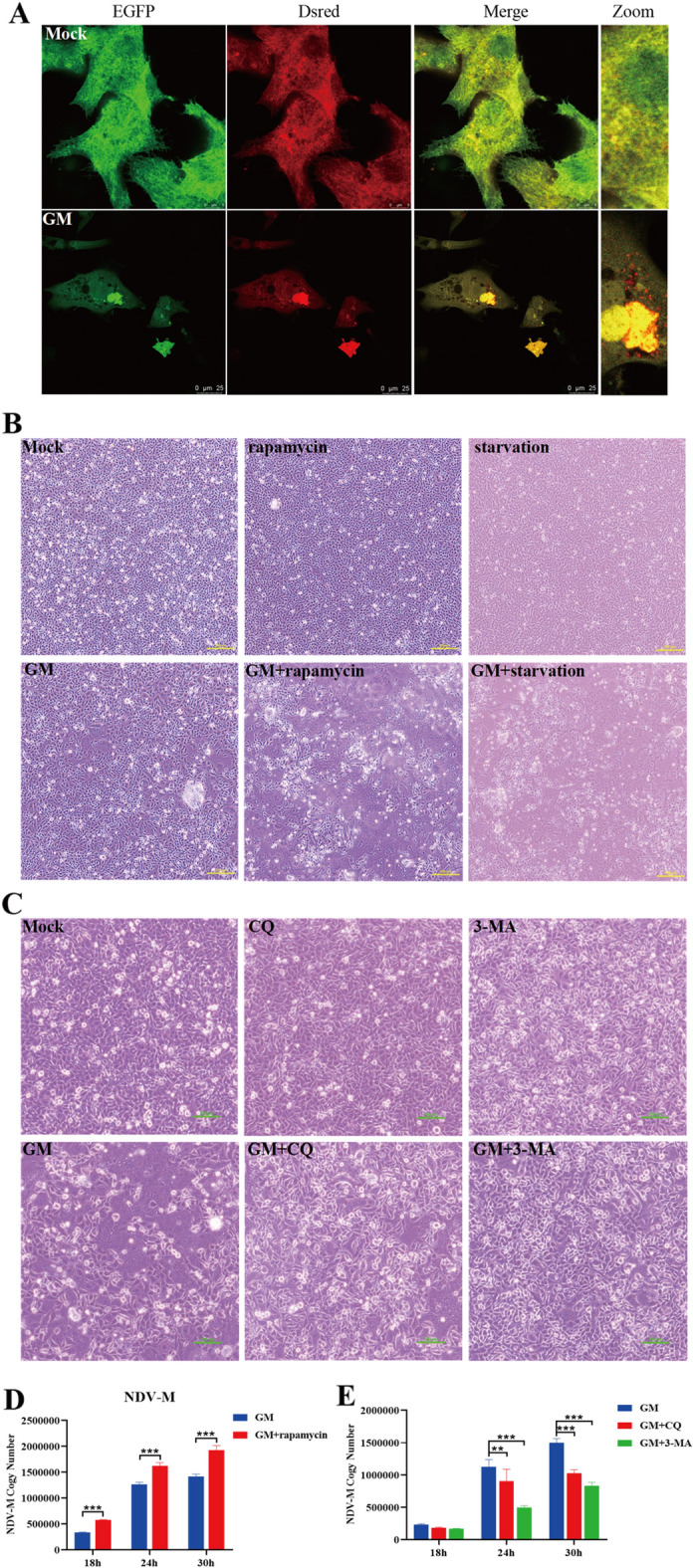
**NDV-induced autophagy promotes cytopathic and viral replication.** DF-1 cells were transfected with Dsred-LC3-EGFP. Twenty-four hours post-transfection, cells were infected with NDV or mock-treated (negative control). **A **Confocal fluorescence microscopy shows the distribution of LC3 in DF-1 cells. **B** and **C** Pathological images of DF-1 cells that underwent starvation or were rapamycin, CQ, 3-MA, or mock-treated, followed by NDV infection at an MOI of 1 for 24 h. Bars, 200 μm (**A**) and 100 μm (**B**). **D** and **E** Transcription levels of the NDV M gene in NDV-infected DF-1 cells measured by qRT-PCR. Similar results were obtained from three independent experiments. Data were expressed as mean ± SD. ^*^P ≤ 0.05; ^**^P ≤ 0.01; ^***^P ≤ 0.001. NDV: Newcastle disease virus, CQ: chloroquine, 3-MA: 3-Methyladenine,  MOI: Multiplicity of infection.

To determine if NDV infection induces autophagy, we used the Dsred-LC3-EGFP dual fluorescent reporter system to investigate the presentation of LC3 protein in the lysosomes of NDV-infected DF-1 cells. The dual fluorescent reporter system has a segment of the LC3 sequence inserted between the Dsred and EGFP dual fluorescent genes to anchor red and green fluorescence to the LC3 protein simultaneously. In the absence of autophagy, the LC3 protein appears yellow due to the combination of Dsred and EGFP fluorescence. In NDV-infected DF-1 cells, LC3 is presented in the lysosomes when autolysosomes are formed. The acidic environment of lysosomes quenches the EGFP fluorescence (green), leaving behind only the red fluorescence from Dsred. The LC3 protein was red, indicating that NDV could induce autophagy in DF-1 cells (Figure 1A).

To explore the effect of NDV-induced autophagy on NDV replication, we promoted autophagy by pretreating DF-1 cells with rapamycin or starvation before NDV infection. As shown in Figure 1B, cytopathic effects of NDV infection were apparent in DF-1 cells 24 h post-infection (hpi). However, pretreatment with rapamycin or starvation enhanced the cytopathic effects of NDV infection. In contrast, pretreatment with CQ or 3-MA, autophagy inhibitors decreased the cytopathic effects of NDV infection (Figure 1C). These findings suggest that autophagy can exacerbate NDV-induced cytopathic effects in DF-1 cells.

To further confirm that NDV-induced autophagy enhances viral replication, we measured the mRNA levels of the NDV M gene in DF-1 cells at different time points following NDV infection. The mRNA level of the NDV M gene in NDV-infected DF-1 cells was significantly upregulated with rapamycin pretreatment (Figure 1D) and significantly down-regulated upon pretreatment with CQ or 3-MA (Figure 1E). The above results indicate that NDV-induced autophagy could enhance cytopathic and viral replication.

### NDV-induced autophagy promotes the expression of inflammatory cytokines

**Figure 2:**
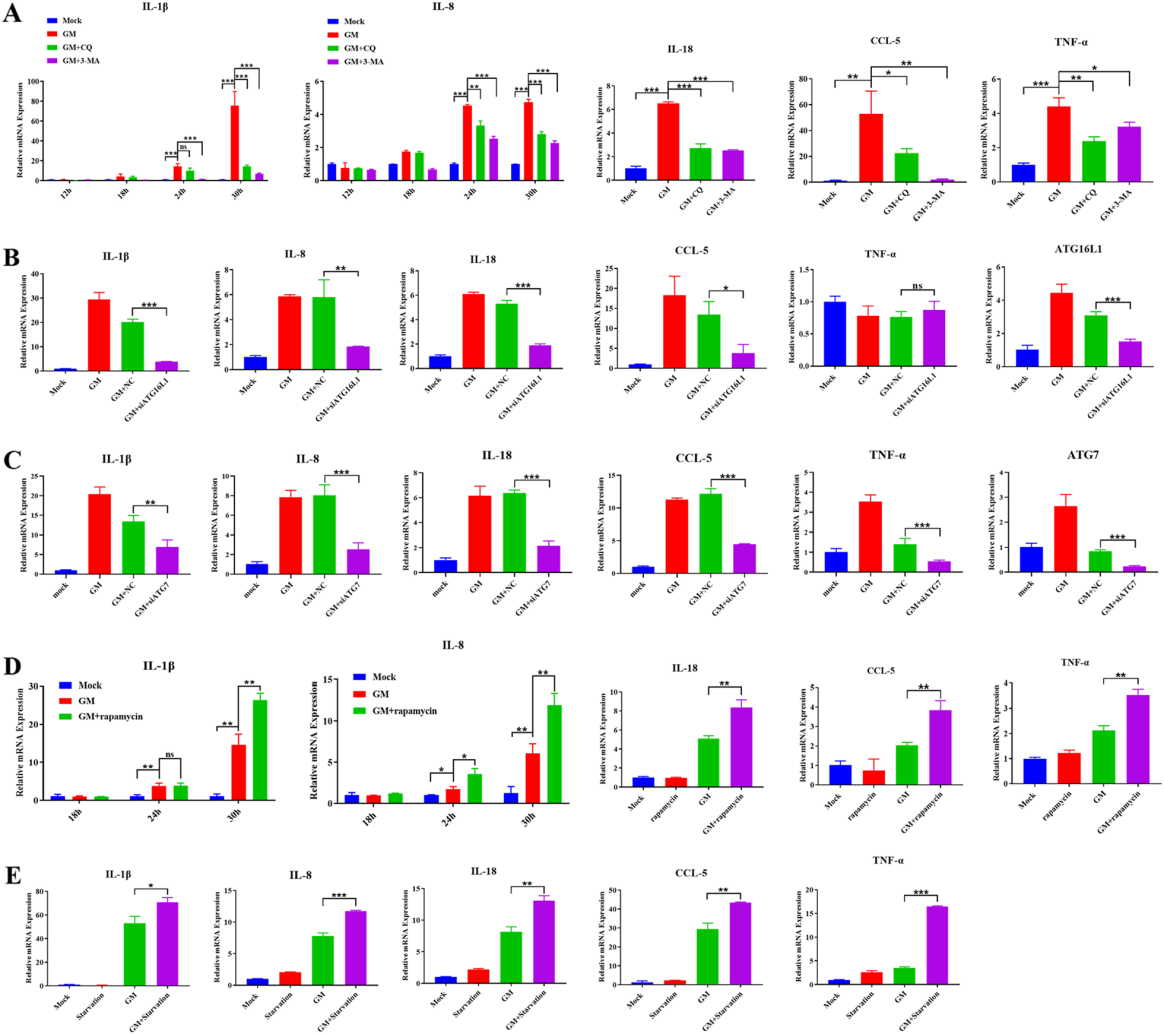
**Effect of NDV-induced autophagy on the transcription of inflammatory cytokines**. **A** The mRNA transcription levels of IL-1β, IL-8, IL-18, CCL-5, and TNF-α after autophagy inhibitors treatment. **B** and **C** The mRNA transcription levels of IL-1β, IL-8, IL-18, CCL-5, TNF-α, ATG16L1 and ATG7 after ATG16L1 or ATG7-specific siRNAs treatment. **D **The mRNA transcription levels of IL-1β, IL-8, IL-18, CCL-5, and TNF-α after autophagy promoter treatment. **E** The mRNA transcription levels of IL-1β, IL-8, IL-18, CCL-5, and TNF-α after starvation treatment. Similar results were obtained from three independent experiments. Data were expressed as mean ± SD. ^*^P ≤ 0.05; ^**^P ≤ 0.01; ^***^P ≤ 0.001. NDV: Newcastle disease virus, CQ: chloroquine, 3-MA: 3-Methyladenine, ATG16L1: autophagy-related protein 16 like protein 1, MOI: Multiplicity of infection.

NDV induces a severe inflammatory response, further aggravating the cytopathic effects of NDV infection [22, 23]. Several studies have shown a close relationship between autophagy and inflammation [8, 24, 25, 29]. To explore the effect of NDV-induced autophagy on inflammation, we assessed the effects of autophagy inhibition on the transcriptional levels of IL-1β and IL-8 in NDV-infected DF-1 cells using qPCR. A significant increase was seen in the transcription levels of IL-1β and IL-8 after 24 hpi. However, pretreatment with CQ or 3-MA significantly reduced the NDV-induced mRNA levels of IL-1β and IL-8 (Figure 2A). Most significant changes in the mRNA levels of IL-18, CCL-5, and TNF-α were seen at 30 hpi, which decreased upon pretreatment with autophagy inhibitors, CQ, or 3-MA (Figure 2A).

ATG16L1 and ATG7 is critical for autophagy initiation [3]. DF-1 cells transfected with siRNAs targeting ATG16L1 or ATG7 exhibited a significant decrease in the transcription levels of various inflammatory cytokines, in response to NDV infection compared to cells transfected with scrambled siRNAs (Figures 2B, C). The results indicate that inhibition of autophagy significantly inhibits the expression of inflammatory cytokines during NDV infection.

To further investigate the effect of NDV-induced autophagy on inflammation, we assessed the mRNA levels of inflammatory cytokines in DF-1 cells that underwent either rapamycin treatment (Figure 2D) or starvation (Figure 2E) before NDV infection. The mRNA levels of all inflammatory cytokines were significantly upregulated in response to NDV infection, which further increased upon pretreatment with rapamycin (Figure 2D) or starvation (Figure 2E). These results suggested that NDV-induced autophagy can promote the expression of inflammatory cytokines.

### NDV-induced autophagy activates NLRP3/Caspase-1 inflammasomes

**Figure 3:**
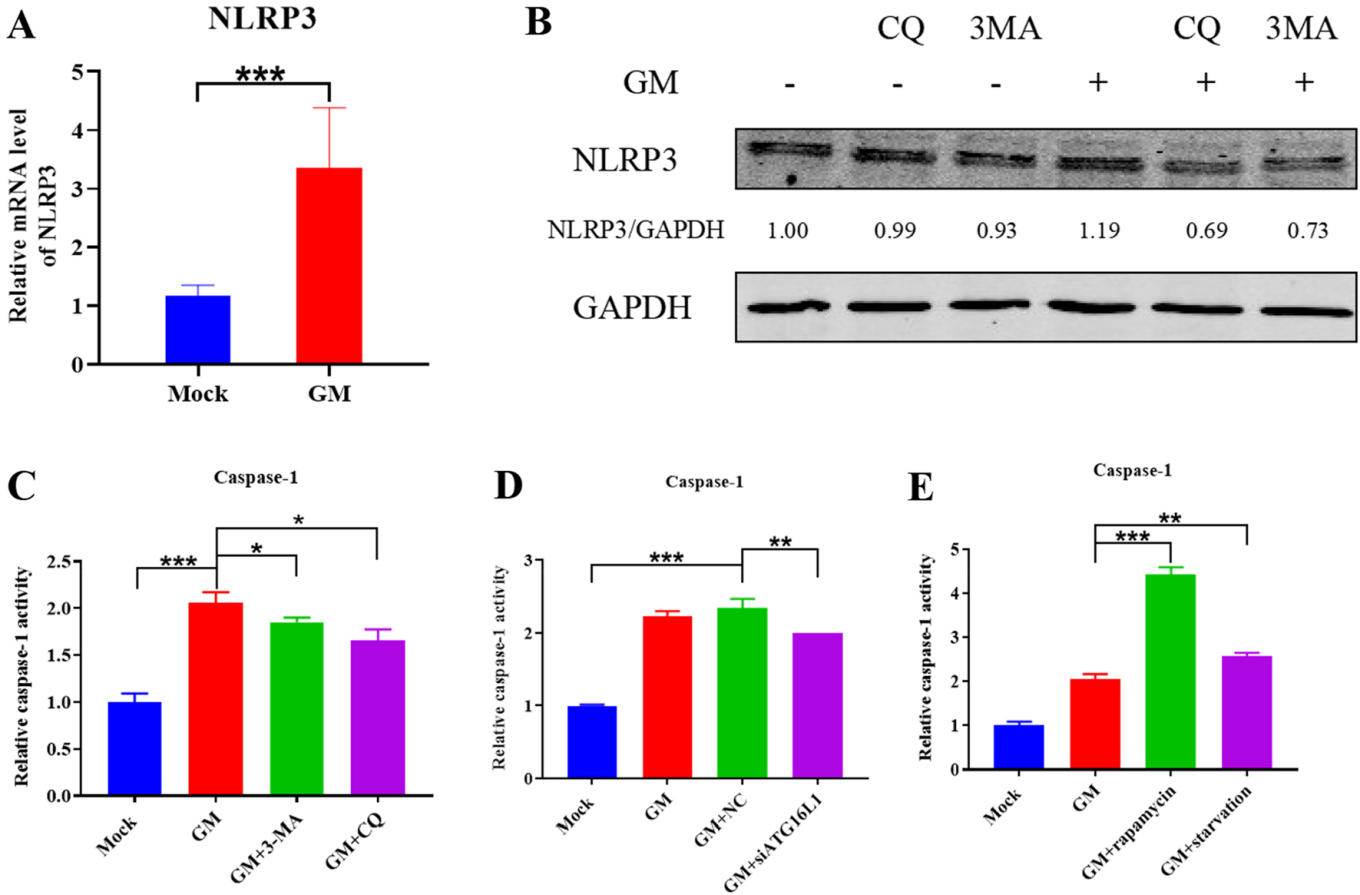
**Effect of NDV-induced autophagy on NLRP3 inflammasome activation.** **A** The mRNA transcription levels of NLRP3 in NDV-infected DF-1 cells. **B** Western blot analysis of NLRP3 protein expression in NDV-infected DF-1 cells treated with CQ or 3-MA. GAPDH was used as a protein loading control. The effect of NDV-induced autophagy on caspase-1 activation was examined in NDV-infected DF-1 cells treated with **C** CQ and 3-MA, **D** ATG16L1-specific siRNAs, and **E** rapamycin and starvation. Shown are the ratios of Caspase-1 activation, measured 30 h after NDV infection. Similar results were obtained from three independent experiments. Data were expressed as mean ± SD. ^*^P ≤ 0.05; ^**^P ≤ 0.01; ^***^P ≤ 0.001. NDV: Newcastle disease virus, CQ: chloroquine, 3-MA: 3-Methyladenine, ATG16L1: autophagy-related protein 16 like protein 1, NLRP3: NOD-like receptor thermal protein domain associated protein 3.

NOD-like receptor thermal protein domain associated protein 3 (NLRP3) inflammasome is crucial for the maturation and production of IL-1β [34–36]. It mainly comprises pro-cysteine-containing aspartate-specific protease-1 (pro-Caspase-1), apoptosis-associated speck-like protein containing a CARD (ASC), and NLRP3 [34]. To determine whether NDV-induced autophagy could affect inflammation through the NLPR3 inflammasome, we examined the mRNA transcription and protein expression levels of NLRP3 in NDV-infected DF-1 cells, and both the transcription and protein expression levels of NLRP3 were up-regulated (Figures 3A and B). In addition, western blot analysis showed a significant decrease in NLRP3 protein expression following the inhibition of autophagy (Figure 3B), indicating that NDV-induced autophagy activates the NLRP3 inflammasome.

Caspase-1 is a protease responsible for converting the key proinflammatory cytokines IL-1β and IL-18 from inactive precursors to active molecules [36]. We studied the effect of NDV-induced autophagy on Caspase-1 activation by using inhibitors (CQ, 3-MA, and ATG16L1-specific siRNA) or activators (rapamycin and starvation) of autophagy in NDV-infected DF-1 cells. Caspase-1 was significantly activated following NDV infection. However, treatment with CQ or 3-MA decreased Caspase-1 activation compared to no treatment (Figure 3C). Similar results were obtained upon ATG16L1-specific siRNAs treatment (Figure 3D). In contrast, treatment with rapamycin or starvation increased the Caspase-1 activity significantly (Figure 3E), suggesting a likely positive relationship between Caspase-1 activation and NDV-induced autophagy. These results suggest that NDV-induced autophagy can activate NLRP3/Caspase-1 inflammasomes.

### NDV-induced autophagy affects the expression of inflammatory cytokines through the p38/MAPK pathway

**Figure 4:**
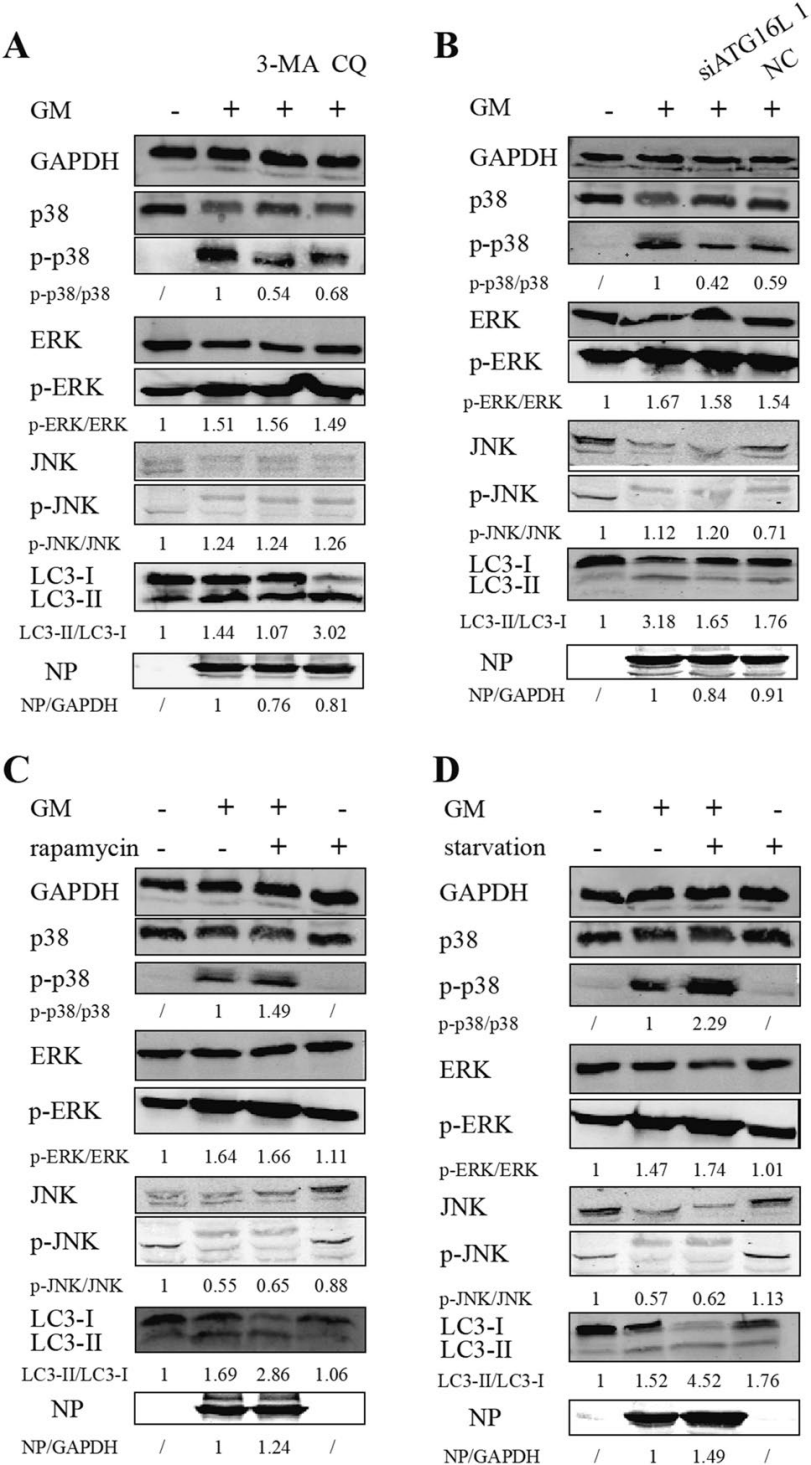
**Effect of NDV-induced autophagy on activation of ERK1/2, JNK, and p38/MAPK pathways, the conversion of LC3-I to LC3-II, and NDV-NP protein expression**. Treatment of NDV-infected DF-1 cells with **A** CQ, 3-MA, and **B** ATG16L1-specific siRNAs led to a decrease in phospho-p38 levels, while no significant changes were seen in the phospho-ERK and phospho-JNK levels, compared cells treated with GM alone. Treatment with (**C**) rapamycin and **D** starvation resulted in a significant increase in the levels of phospho-p38, with no significant changes in the levels of phospho-ERK and phospho-JNK. The bottom blots in each panel show the conversion of LC3-I to LC3-II and the expression of NDV-NP protein in NDV-infected DF-1 cells treated with autophagy inhibitors or promoters. GAPDH is used as a protein loading control. NDV: Newcastle disease virus, CQ: chloroquine, 3-MA: 3-Methyladenine, ATG16L1: autophagy-related protein 16 like protein 1.

The formation of autophagosomes in cells is accompanied by the conversion of LC3-I to LC3-II in the cytoplasm. After the autophagosome binds to lysosomes, a part of the LC3-II is degraded in the autophagosome [3]. As an inhibitor of lysosomes, CQ can effectively block the generation of autolysosomes but does not prevent the conversion of LC3-I to LC3-II, leading to the accumulation of LC3-II [37]. In contrast, 3-MA, an inhibitor of class III PI3K, effectively inhibits the formation of autophagosomes and indirectly inhibits the conversion of LC3-I into LC3-II, leading to a reduction in LC3-II [37]. We studied the changes in LC3 and NDV-NP protein expression in NDV-infected DF-1 cells in response to autophagy inhibitors and promoters. The conversion of LC3-I to LC3-II was apparent at 30 hpi. Treatment with CQ led to further accumulation of LC3-II (Figure 4A), while treatment with 3-MA or ATG16L1-specific siRNAs decreased LC3-II expression (Figures 4A, B), indicating that NDV can induce autophagy, while CQ and 3-MA can effectively inhibit autophagy. In addition, the conversion of LC3-I to LC3-II was significantly reduced by rapamycin treatment (Figure 4C) or starvation (Figure 4D).

The expression of NDV-NP protein in NDV-infected DF-1 cells decreased in response to autophagy inhibitors or ATG16L1-specific siRNAs, compared to the untreated NDV-infected cells (Figures 4A, B) and increased in response to autophagy promoters (Figures 4C and D), consistent with the mRNA levels of NDV-M at 30 hpi.

Several studies have shown that in addition to the NLRP3 inflammasome, various signaling pathways regulate inflammatory cytokines, including ERK1/2, JNK, and p38/MAPK [23, 38, 39]. We assessed the expression of essential proteins in these pathways to explore other mechanisms involved in regulating the inflammatory response by NDV-induced autophagy. The phospho-p38 levels were significantly decreased in the CQ + GM, 3-MA + GM, and siATG16L1 + GM groups, compared to the GM group, while phospho-ERK and phospho-JNK levels showed no significant differences (Figures 4A and B). In addition, the phospho-p38 expression was significantly increased in the rapamycin + GM and starvation + GM groups, while the levels of phospho-ERK and phospho-JNK showed no significant differences (Figures 4C, D).

### NDV infection triggers mitochondrial damage and mitophagy in DF-1 cells

**Figure 5:**
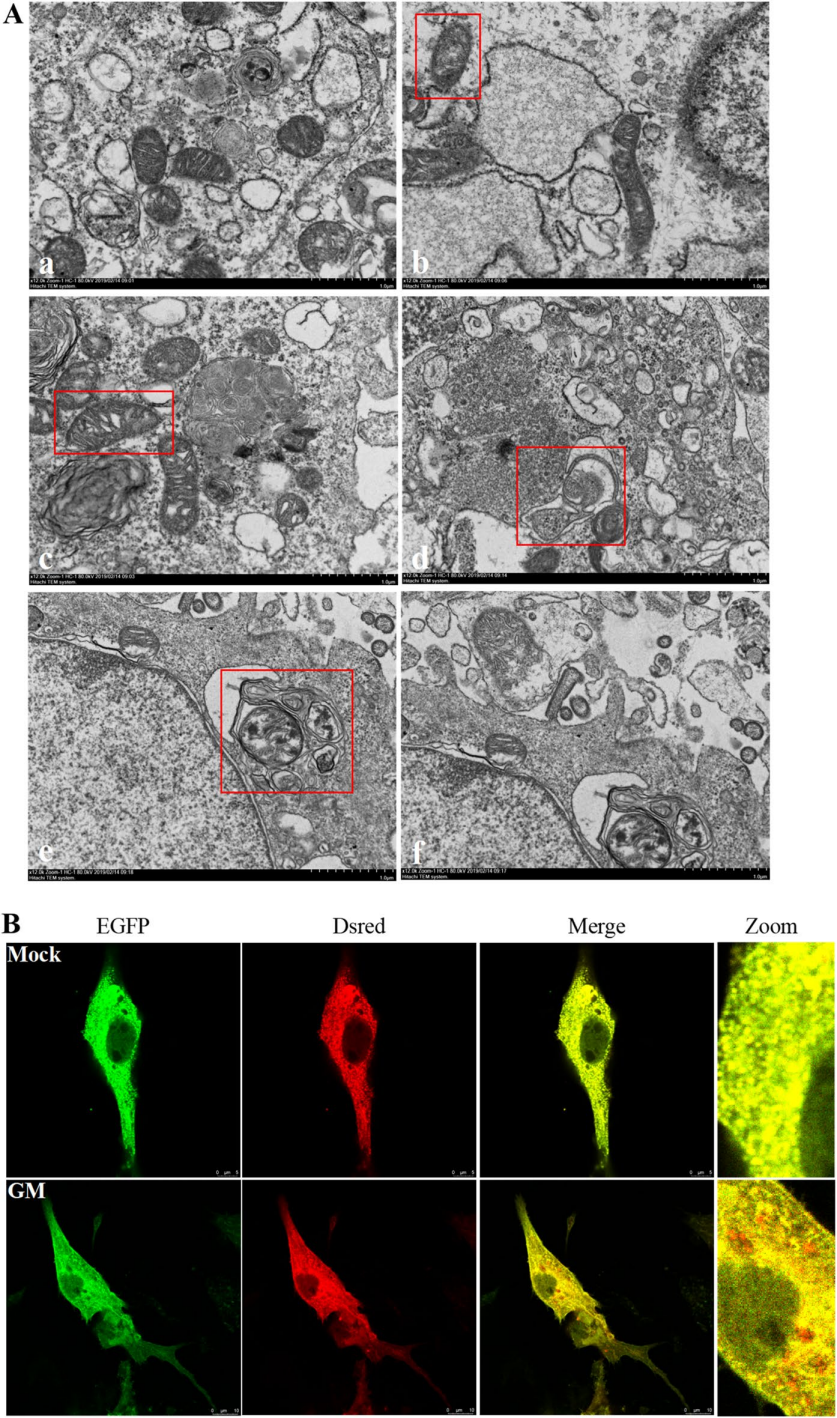
**NDV infection triggers mitochondrial damage and mitophagy in DF-1 cells.** **A** TEM findings. (panel a) DF-1 cells were mock-treated as a negative control. Cells were infected with NDV at an MOI of 1 for (panels b and c) 9 h, (panel d) 12 h, and (panels e and f) 15 h. Bars, 1 μm (panels a–f). **B** Confocal fluorescence microscopy. DF-1 cells transfected with Dsred-Mito-EGFP were either NDV-infected or mock-treated (negative control). Mitochondrial fluorescence was evaluated 24 h post-infection. NDV: Newcastle disease virus.

Mitochondria form a bridge between autophagy and inflammation [26–28]. Mitophagy is the autophagy of the mitochondria and is visualized by TEM. The mitochondria of healthy DF-1 cells were mostly tubular and spherical, with a clear mitochondrial ridge structure perpendicular to the long axis of mitochondria (Figure 5A, panel a). At 9 hpi, the mitochondria appeared swollen (Figure 5A, panel b). Although a clear ridge structure could be observed, the gap between the ridge structures became more prominent, and the structure was loose (Figure 5A, panel c). At 12 hpi, part of the mitochondrial ridge structure disappeared and became smaller. Mitophagy led to the encapsulation and formation of double-membraned autophagosomes (Figure 5A, panel d). At 15 hpi, autophagosomes fused with monolayer membrane structures to form autolysosomes (Figure 5A, panel e), and the degraded mitochondria could be seen in the autolysosomes (Figure 5A, panel f). These observations confirmed NDV infection-induced mitochondrial damage and mitophagy.

The Dsred-Mito-EGFP dual fluorescent reporter system was used to visualize the presence of mitochondria in the lysosomes. The mitochondria of normal cells appear yellow due to the combination of red and green fluorescence. However, NDV-infected DF-1 cells appear red due to the quenching of EGFP fluorescence after the mitochondria are presented to lysosomes, suggesting that NDV infection causes mitochondrial degradation by lysosomes, leading to mitophagy (Figure 5B).

### Intracellular ROS and mtDNA have little effect on inflammation after NDV infection

**Figure 6:**
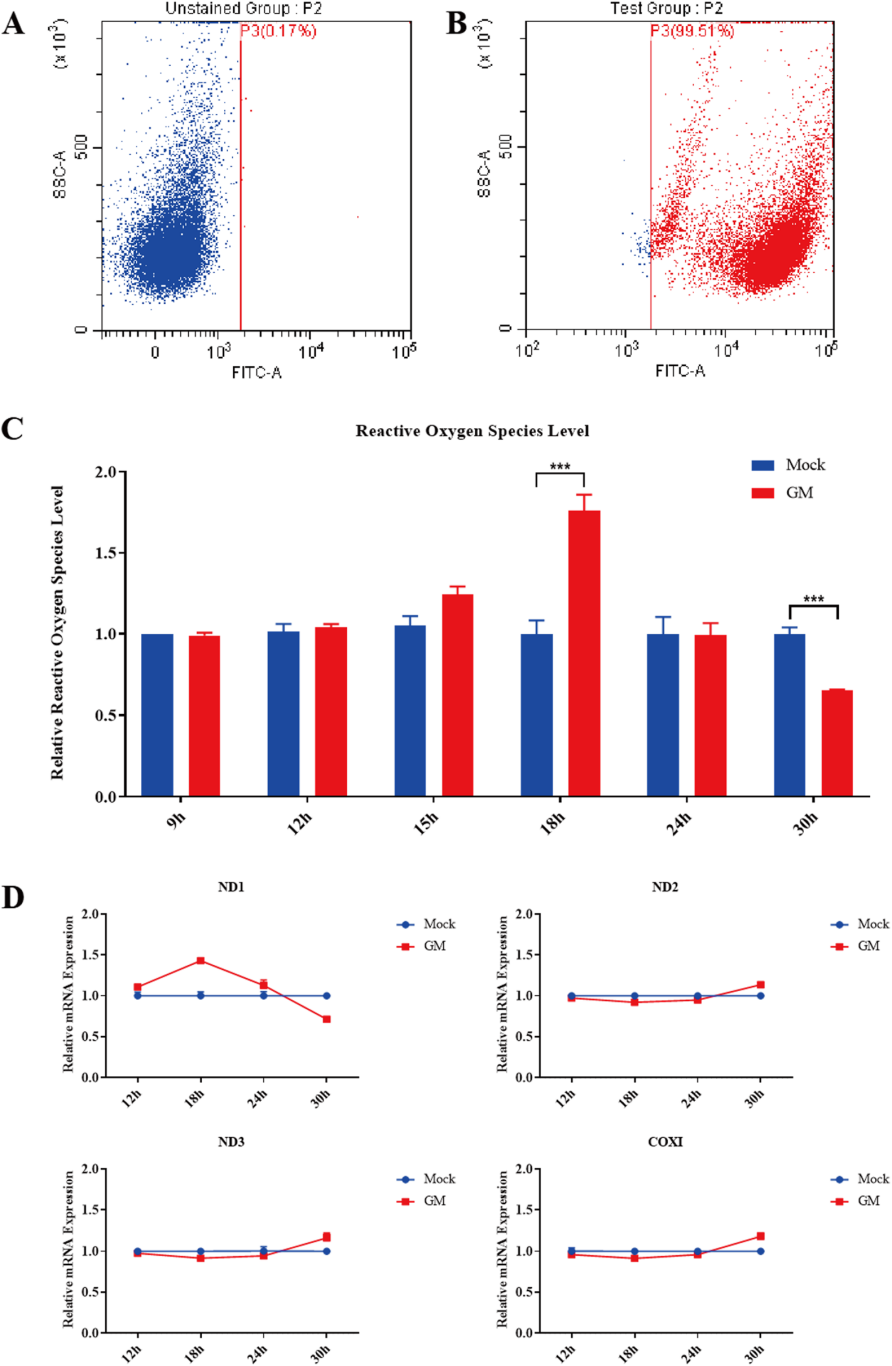
**ROS levels and mtDNA copy number in NDV-infected DF-1 cells.** **A** and **B** DF-1 cells treated with DCFH-DA were assessed for DCF fluorescence by flow cytometry. **C** ROS levels were measured by DCF fluorescence in NDV-infected or mock-treated DF-1 cells. Shown are the relative ratios of ROS levels at indicated time points. **D** Changes in copy number of mitochondrial ND1, ND2, ND3, and COXI genes in NDV-infected DF-1 cells. Similar results were obtained from three independent experiments. Data were expressed as mean ± SD. ^*^P ≤ 0.05; ^**^P ≤ 0.01; ^***^P ≤ 0.001. NDV: Newcastle disease virus, CQ: chloroquine, 3-MA: 3-Methyladenine, MOI: Multiplicity of infection.

DCFH-DA is a cell-permeable fluorescent probe that gets hydrolyzed to DCFH by intracellular esterase upon entering the cell. DCFH cannot pass through the cell membrane, which makes it easy to load the probe into the cell. Reactive oxygen species (ROS) oxidize non-fluorescent DCFH to DCF, emitting green fluorescence. The intensity of the green fluorescence is proportional to the ROS content in the cell. DCF fluorescence was measured by flow cytometry (Figures 6A, B). ROS levels were increased in DF-1 cells at 18 hpi, followed by a decrease in the late stage of NDV infection (Figure 6C).

Total DNA was extracted from DF-1 cells, and four genes of mtDNA were quantified using qPCR to determine the effect of NDV infection on mtDNA copy number in cytoplasm, including three NADH dehydrogenase subunits (ND-1, ND-2, ND-3) and one cytochrome C oxidase subunit I (COXI). As shown in Figure 6D, no significant differences were seen in the copy numbers of the four mitochondrial genes at 30 hpi, indicating that mtDNA was not released heavily from mitochondria after NDV infection. These findings suggest that intracellular ROS and mtDNA have little effect on the expression of inflammatory cytokines during NDV infection.

## Discussion

Autophagy is a part of the host defense system, leading to the degradation of invading microorganisms by delivering them to the lysosomes [3]. However, although this process is a cellular defense mechanism, some viruses have evolved mechanisms to counter autophagy and promote replication [9]. Previous studies have shown that NDV can induce autophagy in human and avian cells [40, 41]. Sun et al. showed that NDV infection could induce autophagy response in chicken tissues, and NDV utilizes autophagy to promote its own replication [18]. In this study, we confirmed that NDV infection enhances autophagy in chicken cells using a Dsred-LC3-EGFP dual fluorescent reporter system and by western blotting. In addition, NDV-induced autophagy enhanced cytopathic and viral replication. However, the mechanism by which NDV utilizes autophagy to promote viral replication after invading host cells has not been clarified. According to current studies, autophagy can regulate inflammatory signaling through various pathways to promote the inflammatory response [8, 24, 25, 29]. Therefore, it is believed that NDV-induced autophagy may exacerbate the cytopathic effects and promote viral replication by influencing inflammatory responses.

The inflammatory response is an essential part of innate immunity and plays an indispensable role in the host defense mechanism, which includes cytokines and receptors of the interleukin-1 family. However, a hyper inflammatory response may cause damage to the body instead [19, 20]. NDV infection intensifies the host inflammatory response, affecting the pathology of the lymphatic tissues and organs, leading to high mortality in the host [21]. We have previously shown that neutralizing IL-1β reduced NDV-driven morbidity and mortality, suggesting that NDV-induced violent inflammatory responses are an important cause of poultry death [22, 23]. In this study, we explored the effect of NDV-induced autophagy on the inflammatory response. We found a likely positive relationship between autophagy and the expression of some essential inflammatory cytokines, such as IL-1β, IL-18, IL-8, CCL-5, and TNF-α, suggesting that NDV-induced autophagy can promote the expression of inflammatory cytokines, which may further enhance NDV-induced inflammatory response.

Autophagy and inflammation are two fundamental processes that initiate the body’s defense response. With a better understanding of the immune system, autophagy and related pathways have been integrated into complex signaling networks that coordinate cellular defense strategies [9, 29]. We previously determined that NDV induces IL-1β expression through p38, JNK/MAPK pathways, and NLRP3/Caspase-1 inflammasomes [22, 23]. In this study, further investigating the effects of NDV-infected autophagy on NLRP3 inflammasome and inflammatory pathways, we found that NLRP3 protein expression decreased significantly following treatment with autophagy inhibitors. In addition, Caspase-1 activity was positively correlated with the degree of autophagy, indicating that NDV-induced autophagy can stimulate NLRP3 inflammasome. Activation of inflammasomes prompts ASC, leading to the cleavage of pro-Caspase-1 into active Caspase-1, which in turn promotes the maturation of IL-1β and IL-18, inducing inflammation.

MAPK pathway plays an important regulatory role in the occurrence and development of inflammatory response, and many viruses can regulate the expression of inflammatory factors through the MAPK pathway. Coinfection of porcine circovirus 2 and pseudorabies virus can promote the activation of p38, JNK/MAPK pathways to enhance the expression of various inflammatory cytokines [42]. In lung fibroblasts, p38/MAPK signaling inhibition significantly suppresses parainfluenza virus-induced IL-1β expression [31]. In this study we determined that NDV-induced autophagy could activate p38/MAPK pathway and the p38 phosphorylation was positively correlated with the mRNA transcription levels of inflammatory cytokines. Coupled with our previous findings that NDV-induced IL-1β expression was significantly decreased with p38 MAPK inhibitor treatment in DF-1 cells [23], we identified that NDV-induced autophagy can promote the expression of inflammatory cytokines through p38/MAPK pathway. In addition to its role in inflammatory response, p38 MAPK can be activated during NDV infection to benefit viral mRNA translation via interaction of the viral NP protein and host eIF4E [43]. Moreover, p38 MAPK is involved in NDV-induced A549 cells death [38]. Some viruses, such as SARS-CoV-2, have been shown to inhibit the PI3K/AKT/mTOR pathway by upregulating intracellular ROS levels to promote the autophagic response, triggering inflammatory responses and apoptosis in infected cells [44]. In future studies, we will examine other critical pathways to better understand the regulatory mechanisms between autophagy and inflammation.

Mitochondria are the most essential and sensitive cellular organelles and play a crucial role in apoptosis, necrosis, autophagy, stress regulation, and innate immunity [45]. ROS is a highly active oxygen-containing species, mainly produced in the mitochondrial respiratory chain [46]. Damaged mitochondria produce excessive ROS and induce oxidative mtDNA (ox-mtDNA) generation, which is either repaired by DNA glycosylase OGG1 or escapes through the mitochondrial permeability transition pore (mPTP) [47]. ROS from mitochondria can induce autophagy and activate NLRP3 inflammasome and multiple inflammation-related transcription factors with mtDNA [48]. When mitochondria are damaged, they discharge their oxidized and cleaved mtDNA into cytosolic solutions, which subsequently enter the bloodstream, causing inflammation. Studies have shown that when mtDNA is released outside the mitochondria, it can activate toll-like receptor 9 (TLR-9), leading to the activation of NF-κB, MAPK, and NLRP3 inflammasome signaling pathways [47, 49]. However, cells have developed mitophagy, a strategy to cope with mitochondrial abnormalities. Mitophagy is an atypical autophagy process that selectively eliminates redundant or damaged mitochondria, which helps regulate the number of mitochondria and maintain their normal function. The damaged mitochondria can be eliminated by selective autophagy, preventing the production of more ROS and mtDNA and inhibiting the activation of the NLRP3 inflammasome [4, 50]. In this study, we found an increase in the intracellular ROS level during the early stage of NDV infection followed by a decrease and lower than the normal levels at 30 hpi. These findings suggested mitophagy may be activated after mitochondrial abnormalities to dispose of excess ROS and maintain mitochondrial homeostasis. Quantitation of mtDNA showed that NDV infection did not cause a large amount of mtDNA to escape from mitochondria. This further confirmed the hypothesis that mitophagy could clear damaged mitochondria and reduce the activation of inflammation by ROS and mtDNA leakage. These results, combined with the significantly increased expression of inflammatory cytokines at 30 hpi, indicated that ROS and mtDNA are not the primary factors driving inflammation in NDV-infected DF-1 cells. Some studies have also shown that mtDNA escaping from mitochondria activates inflammation through the cGAS-STING pathway [51]. However, the regulatory relationship between mitophagy and inflammation after NDV infection still requires further investigation, which will expand our understanding of the interaction between mitophagy and inflammation.

The present study reveals the regulatory effect of NDV-induced autophagy on inflammatory response. However, determining whether inflammation has a feedback and regulatory effect on autophagy, and verifying how inflammation regulates NDV-induced autophagy in chickens warrant further investigations. This will expand our understanding of the interaction between autophagy and inflammation during NDV infection.

In conclusion, we demonstrate that NDV-induced autophagy enhances inflammation through the NLRP3/Caspase-1 inflammasomes and p38/MAPK pathway. These results provide insights into the molecular mechanisms underlying the regulatory relationship between autophagy and inflammation induced during NDV replication.

## Supplementary information

**Additional file 1**  **Information of newly designed primers and GAPDH**. PCR efficiency calculation with R^2^ value and linear dynamic range of newly designed primers. Melt curves of newly designed primers. Amplification curves of GAPDH in DF-1 cells that under went GM infection, siRNAs transfection, siRNA transfection with GM infection, or mock treatment. Melt curves of GAPDH in DF-1 cells that under went GM infection, siRNAs transfection, siRNA transfection with GM infection, or mock treatment.

## Data Availability

The datasets during and/or analyzed during the current study available from the corresponding author on reasonable request.
