# Supplementary figures and images for: NDV-induced autophagy enhances inflammation through NLRP3/Caspase-1 inflammasomes and the p38/MAPK pathway
Source: Vet Res. 2023 Jun 5;54:43. doi: 10.1186/s13567-023-01174-w (PMC10240457; doi:10.1186/s13567-023-01174-w)

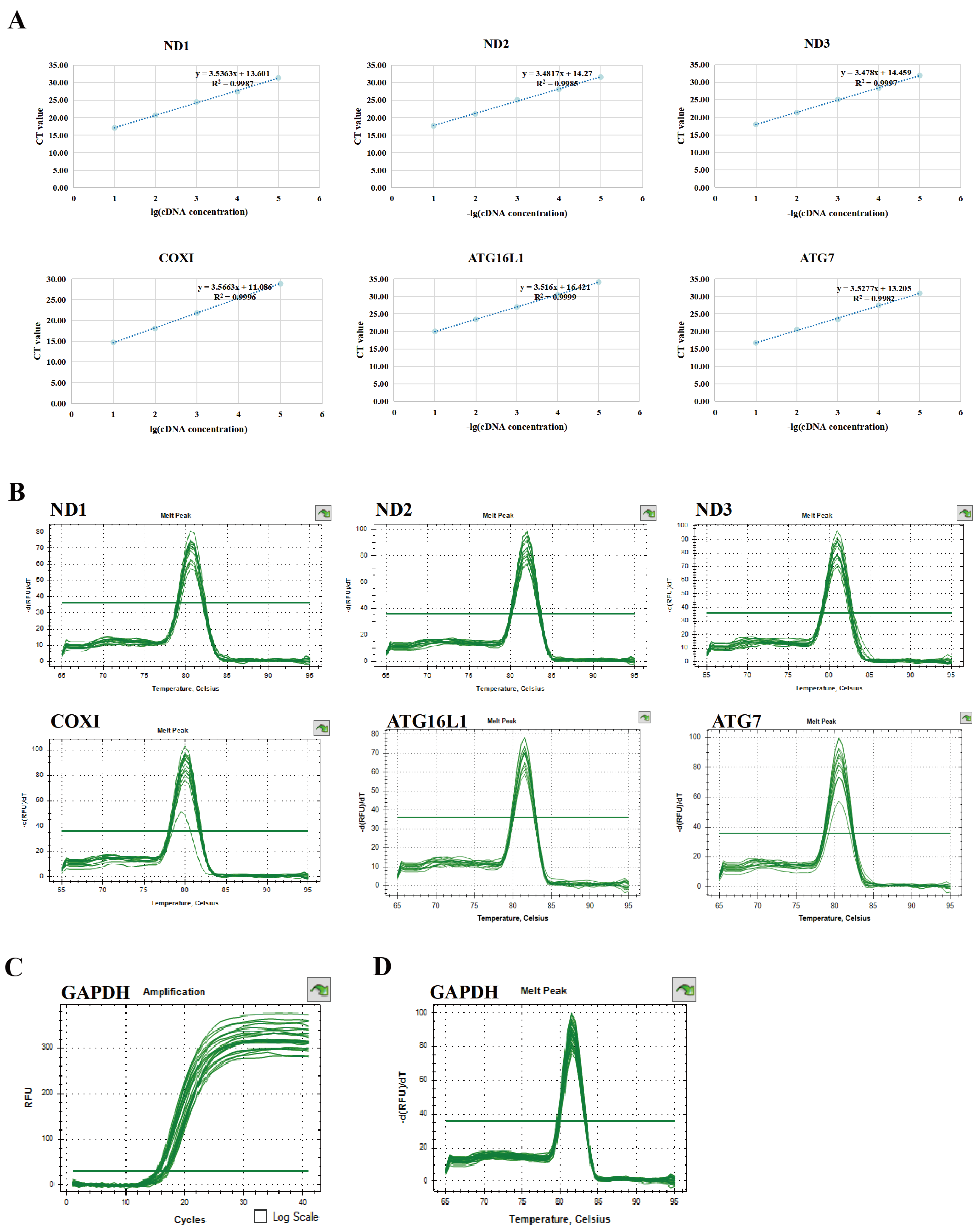

Supplement: Supplementary file 1 — Additional file 1 Information of newly designed primers and GAPDH. PCR efficiency calculation with R2 value and linear dynamic range of newly designed primers. Melt curves of newly designed primers. Amplification curves of GAPDH in DF-1 cells that under went GM infection, siRNAs transfection, siRNA transfection with GM infection, or mock treatment. Melt curves of GAPDH in DF-1 cells that under went GM infection, siRNAs transfection, siRNA transfection with GM infection, or mock treatment. [file 13567_2023_1174_MOESM1_ESM.docx]
